# Intra- and interobserver variability of whole-tumour apparent diffusion coefficient measurements in nephroblastoma: a pilot study

**DOI:** 10.1007/s00247-015-3354-4

**Published:** 2015-05-08

**Authors:** Annemieke S. Littooij, Paul D. Humphries, Øystein E. Olsen

**Affiliations:** Department of Radiology, University Medical Centre Utrecht, Heidelberglaan 100, 3584 CX Utrecht, The Netherlands; Department of Radiology, Great Ormond Street Hospital for Children, London, UK; Department of Radiology, University College London Hospital, London, UK

**Keywords:** Apparent diffusion coefficient, Children, Diffusion-weighted imaging, Magnetic resonance imaging, Nephroblastoma, Observer variation, Wilms tumour

## Abstract

**Background:**

The apparent diffusion coefficient (ADC) is potentially useful for assessing treatment response in nephroblastoma (Wilms tumour). However the precision of ADC measurements in these heterogeneous lesions is unknown.

**Objective:**

To assess intra- and interobserver variability of whole-tumour ADC measurements in viable parts of nephroblastomas at diagnosis and after preoperative chemotherapy.

**Materials and methods:**

We included children with histopathologically proven nephroblastoma who had undergone MRI with diffusion-weighted imaging before and after preoperative chemotherapy. Three independent observers performed whole-tumour ADC measurements of all lesions, excluding non-enhancing areas. One observer evaluated all lesions on two occasions. We performed analyses using Bland–Altman plots and concordance correlation coefficient (CCC) calculations with 95% limits of agreement for median ADC, difference between pre- and post-chemotherapy median ADC (ADC shift) and percentage of pixels with ADC values <1.0 × 10^−3^ mm^2^/s.

**Results:**

In 22 lesions (13 pretreatment and 9 post-treatment) in 10 children the interobserver variability in median ADC and ADC shift were within the interval of approximately ±0.1 × 10^−3^ mm^2^/s (limits of agreement for median ADC ranged −0.08–0.11 × 10^−3^ mm^2^/s and for ADC-shift −0.11–0.09 × 10^−3^ mm^2^/s). The interobserver variability for percentage of low-ADC pixels was larger and also biased. The calculated CCC confirmed good intra- and interobserver agreement (ρ-c ranging from 0.968 to 0.996).

**Conclusion:**

Measurements of whole-tumour ADC values excluding necrotic areas seem to be sufficiently precise for detection of chemotherapy-related change.

## Introduction

The monitoring of oncological treatment response with cross-sectional imaging is traditionally based on tumour size assessment, but change in size does not always correspond to actual biological response [1–3]. Increase in volume can occur in well-responding tumours, e.g., during differentiation. On the other hand, tumours that shrink may still contain considerable volumes of viable tumour, e.g., blastema and anaplastic elements of nephroblastoma. Therefore, imaging parameters beyond those that estimate overall tumour volume are desirable.

MRI is the preferred modality for imaging renal tumours in children because it provides high soft-tissue contrast and offers anatomical and quantitative information without the use of ionising radiation [4, 5]. Diffusion-weighted MRI (DWI) measures random motion of water protons that is restricted in, for instance, highly cellular lesions. The apparent diffusion coefficient (ADC) is a quantification of the degree of impeded water motion. Hence, it can be utilised as a noninvasive in vivo biomarker. A decrease in cellularity, a common histopathological treatment response, might be detected as increased apparent diffusion at DWI [6]. Specifically, reports in nephroblastoma have suggested that there is a shift towards higher ADC values in tumours that have responded to preoperative chemotherapy.

Unfortunately there is considerable heterogeneity in reported acquisition techniques and methods of ADC measurements [7], and the variability in measurements is not known. In other words, we do not know whether an observed change in ADC truly reflects a biological process rather than a random measurement error. An important step in exploring this is to estimate the variability caused by imaging readers.

Our aim was therefore to quantify the variability among experienced readers and also between reading sessions, using already proposed parameters of the tumour volume ADC distribution: the median ADC at a single scan, and the difference in median ADC between the pre-and post-chemotherapy scans. Because low ADC is associated with high cellularity [7], we also explored the variability in assessment of the percentage of voxels within tumour with low ADC values.

## Materials and methods

### Patients

The research ethics committee of our institution waived the need for ethics approval for this retrospective review. We included 10 consecutive children referred to our tertiary care paediatric hospital during July 2012–July 2013. All had histopathologically proven nephroblastoma. MRI is the modality of choice for formal cross-sectional tumour imaging at our institution and was performed as part of standard clinical care at initial presentation and after preoperative chemotherapy. Exclusion criteria were incomplete MRI study, predominantly cystic lesions, lesions where the largest area on a single slice was <3 cm^2^ or lesions with a total volume of <6 cm^3^. In smaller lesions there is a considerable risk for partial volume effects. All children were treated according to the guidelines of the International Society of Paediatric Oncology (SIOP-RT 2001) [8]: 4 weeks’ preoperative chemotherapy with vincristine and actinomycin D is considered standard therapy for localised tumours. Children with metastases at diagnosis receive 6 weeks of preoperative therapy with three drugs, including doxorubicin [8].

### Magnetic resonance imaging acquisition

Contrast-enhanced MRI of the abdomen including DWI was performed on a 1.5-T scanner (Avanto; Siemens Healthcare, Erlangen, Germany). The imaging protocol consisted of fat-suppressed axial pre- and post-gadolinium T1-W turbo spin-echo, axial and coronal T2-W short tau inversion recovery, and diffusion-weighted imaging (Table 1). Diffusion-weighted sequences were acquired in the axial plane during free-breathing, applying b values of at least 0 s/mm^2^, 50 s/mm^2^, 250 s/mm^2^, 500 s/mm^2^ and 1,000 s/mm^2^ (Table 1). ADC maps were automatically generated by the MRI measurement system.

**Table 1:** Scan parameters at 1.5-T MRI for suspected renal tumour or for imaging of nephroblastoma after neo-adjuvant chemotherapy

Parameter	T2 STIR	T2 SPACE	DWI	T1 pre/post	T1 pre/post
Pulse sequence	2-Ds short tau inversion recovery (STIR) spin echo	3-D turbo spin echo with variable flip angle	2-D single-shot spin echo with spectral fat saturation	2-D turbo spin echo with fat-suppression	2-D turbo spin echo with fat suppression and variable readout directions
Repetition time (ms)	>6,000	>2,000	2,700	400–670	590
Echo time (ms)	62	238	90	17–20	23
Inversion time (ms)	130				
Slice orientation	Coronal and axial	Axial	Axial	Axial	Axial
Slice thickness (mm)	5–6	0.9	6	5–6	7
Slice gap (mm)	0–0.5	0	1.8–2.4	1–3	1.4
Echotrain length	21	89	1	1	9
Acquisition matrix	256 × 194	256 × 255	128 × 96	256 × 154	192 × 192
Receive bandwidth (Hz/pixel)	200	651	1,500	70	300
b values (s/mm^2^)	–	–	At least 0, 50, 100, 250, 500, 1,000	–	–

*DWI* diffusion-weighted imaging, *Post* post-treatment, *Pre* pretreatment, *SPACE* sampling perfection with application of optimized contrasts using different flip angle evolution, *STIR* short tau inversion recovery

Children were awake, sedated or under general anaesthesia, depending on their ability to cooperate. Gadoterate meglumine (Dotarem; Guerbet Laboratories, Roissy, France) was administered at an intravenous dose of 0.05 mmol/kg body weight. All children were screened for contraindications, such as risk factors for nephrogenic systemic fibrosis. Estimation of glomerular filtration rate was performed in children with suspicion of renal disease and after chemotherapy. All children received the intravenous spasmolytic hyoscine butylbromide (Buscopan; Boehringer Ingelheim Limited, Bracknell, UK) at a dose of 0.4 mg/kg body weight to reduce artefacts caused by peristaltic movements of the bowel.

### Image analysis

The anonymised MRI datasets including the ADC maps were transferred to the DICOM software OsiriX version 5.5.2 (Pixmeo SARL, Bernex, Switzerland). Three independent paediatric radiologists (Ø.E.O, P.D.H and A.S.L, with 14, 10 and 5 years of experience with paediatric abdominal MRI, respectively) performed the ADC measurements. The readers were blinded to one another’s results and to histopathology reports. The pretreatment images were available for review during analysis of the post-treatment datasets. To assess the intra-observer variability, one reader (A.S.L.) obtained the measurements twice, with a minimum interval of 4 weeks. The first reading of this reviewer was used to assess the interobserver variability.

### Whole-tumour ADC measurement protocol

**Fig. 1:**
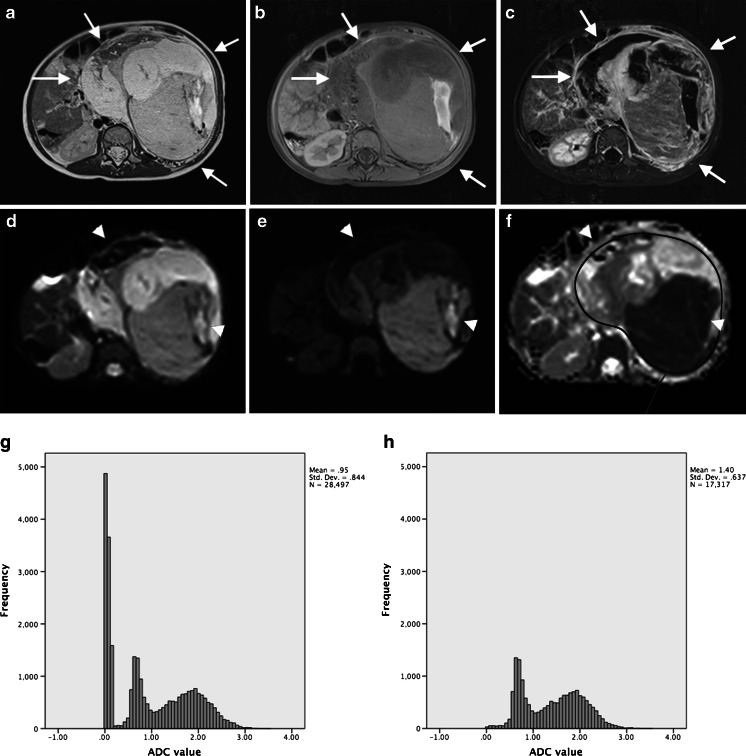
MR images in a 2-year-old girl with left-side nephroblastoma illustrate the importance of including only the viable parts of the tumour in the analysis of ADC distribution. **a** Axial T2-SPACE, (**b**) axial T1-W turbo spin echo and (**c**) subtracted post-contrast axial T1-W MR images show a large heterogeneous tumour (*arrows*) with haemorrhagic and necrotic components arising from the left kidney, consistent with nephroblastoma. **d**–**f** Axial diffusion-weighted images with b values of 0 (**d**) and 1,000 (**e**) and the ADC map (**f**) illustrate the low signal and low ADC within the large necrotic/haemorrhagic components (*arrowheads*). **g**, **h** The whole-tumour ADC histograms (horizontal axis: ADC value [10^−3^ mm^2^/s]) without subtraction (**g**) and with subtraction (**h**) demonstrate the need for excluding the less-enhancing parts of the tumour for ADC analysis. Because of the haemorrhagic/necrotic components within the tumour, the histogram without subtraction shows a high peak ADC value of about 0. This is probably related to susceptibility artefacts and results in incorrect ADC calculations that can skew the median ADC value to a lower value. *ADC* apparent diffusion coefficient, *SPACE* sampling perfection with application of optimized contrasts using different flip angle evolution

Regions of interest (ROIs) were drawn manually on the ADC maps. This was done by each reader for interobserver analysis, and at both readings for intra-observer analysis. Conventional MR images guided the definition of the outline of the tumour at each consecutive tumour-containing slice, excluding peritumoural oedema. To minimise the partial volume effect, we only included the sections where the tumor area was >50% of the adjacent more central slice and where the tumor area was at least 3 cm^2^. We used the pre- and post-gadolinium T1-W images to create a mask that excluded areas of tumour with no or very low enhancement, because these were thought to represent necrosis, haemorrhage or cystic elements. Therefore, we subtracted the pre- from the post-gadolinium T1-W images. We resampled this subtracted dataset against the tumour ADC dataset to obtain similar voxel size and orientation. Further analysis was performed with the image-processing program ImageJ, version 1.47 (National Institutes of Health, Bethesda, MD). The pixels of the subtracted dataset with an enhancement at or above that of the erector spinae muscles were used as a threshold filter for the ADC dataset. The final ADC dataset therefore represented all pixels from the enhancing parts of the tumour. These whole-tumour ADC sets were exported for statistical analysis. The requisite of including only the viable parts of the tumour in the analysis of ADC distribution is exemplified in Fig. 1.

Because not all whole-tumour ADC histograms were normally distributed, the median ADC was chosen to represent the central location of the ADC distribution. Measuring only a mean or median could conceal relevant information. Therefore we also assessed the variability within and between observers for percentage of pixels with low ADC (<1.00 × 10^−3^ mm^2^/s), assuming that lower ADC values are more relevant in assessing treatment response [8, 9]. Last, we assessed the variability in measurements for the difference in median ADC before and after neo-adjuvant chemotherapy.

### Statistical analysis

Intra- and interobserver variability for the three variables (median ADC, percentage of low-ADC pixels, and shift in median ADC during chemotherapy) were analysed according to the method of Bland and Altman with calculation of the 95% limits of agreement [10].

Additionally, the Lin [11] concordance correlation coefficient (CCC) was calculated along with the 95% confidence intervals as another measure of assessing the observer variability.

The Wilcoxon rank sum test was used to compare the pre- and post-treatment median ADC differences for every rater pair to check for potentially significant differences, i.e. rater variability, in pre- and post-treatment lesions. Spearman’s rho was used for assessing any linear relation between the average and the differences for the tested ADC parameters. *P*-values <0.05 were considered statistically significant.

R software (version 3.0.1; R Foundation for Statistical Computing, Vienna, Austria) was used for calculation of the concordance correlation coefficient. All other statistical analyses were executed using Statistical Package for the Social Sciences (version 22.0; IBM, Armonk, NY).

## Results

### Patients

**Fig. 2:**
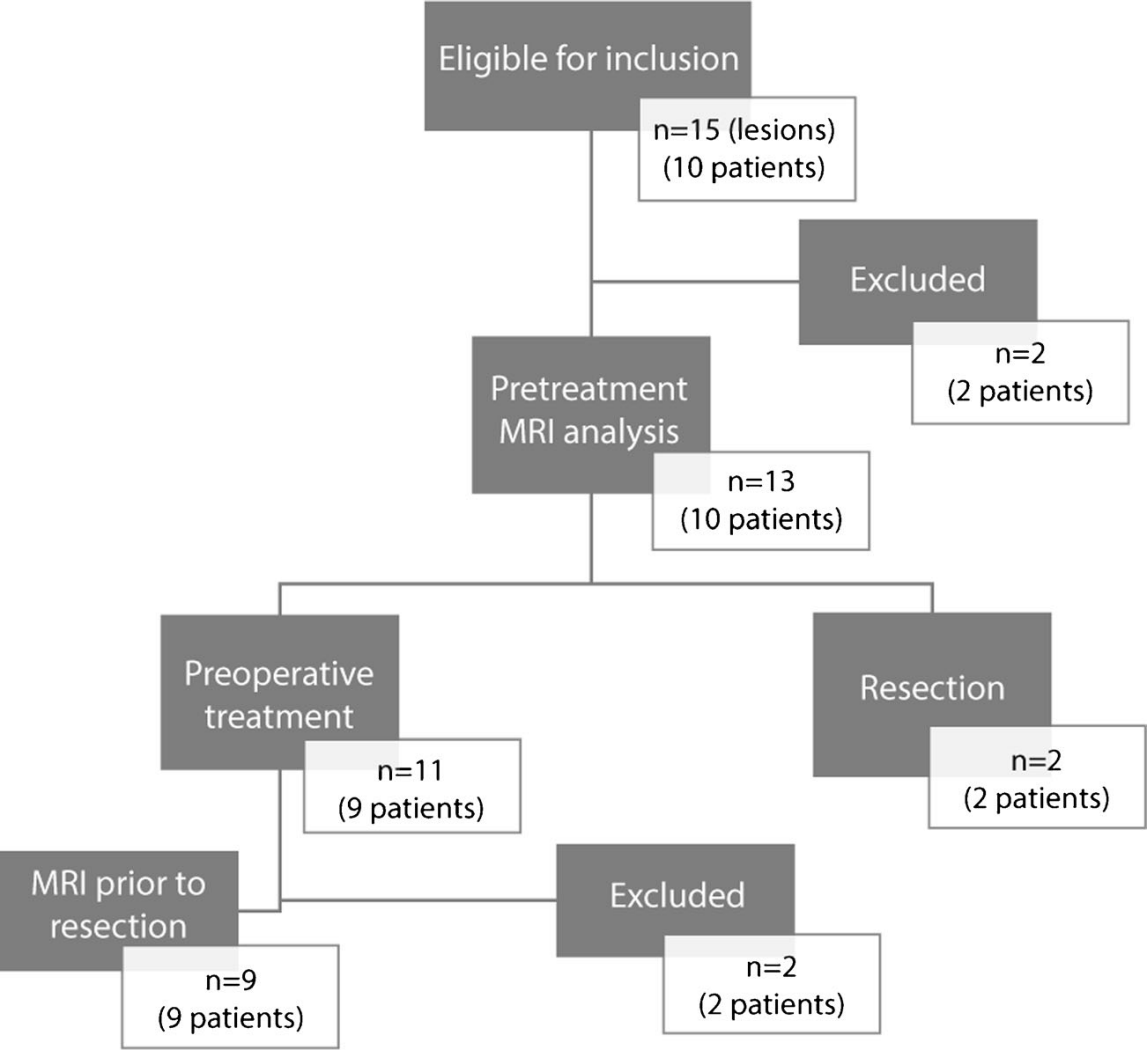
Flow diagram according to standard of reporting diagnostic accuracy studies (STARD). *One child with bilateral disease underwent primary resection of one lesion and preoperative treatment for the contralateral lesion

We included 10 children (mean age 2.7 years, range 0.5–4.5 years). They had a total of 15 histologically proven nephroblastoma lesions. Two lesions were excluded from analysis because of their predominantly cystic nature (Table 2). In 9 of 10 children, complete MRI studies after preoperative therapy were available for analysis. Two additional post-treatment lesions were excluded for the following reasons: too small (*n* = 1, volume 5.5 cm^3^), predominantly cystic (*n* = 1). In total we included 13 pre-treatment (mean volume 323.0 cm^3^, range 7.4–1,157.8 cm^3^) and 9 post-treatment (mean volume 225.3 cm^3^, range 6.2–1,208.1 cm^3^) lesions for final analysis. The flow diagram of standards for reporting diagnostic accuracy studies (STARD) is illustrated in Fig. 2.

**Table 2:** Patient characteristics

Number of children	10
Gender (*n*)	
Male	4
Female	6
Age (*years*)	
Mean ± standard deviation	2.7 ± 1.4
Range	0.5–4.5
Number of lesions (*n*)	13
Pathological subtype	
Regressive	2
Mixed	7
Epithelial	1
Stromal	1
Blastemal	1
Diffuse anaplasia	1

### Intra-observer variability

**Fig. 3:**
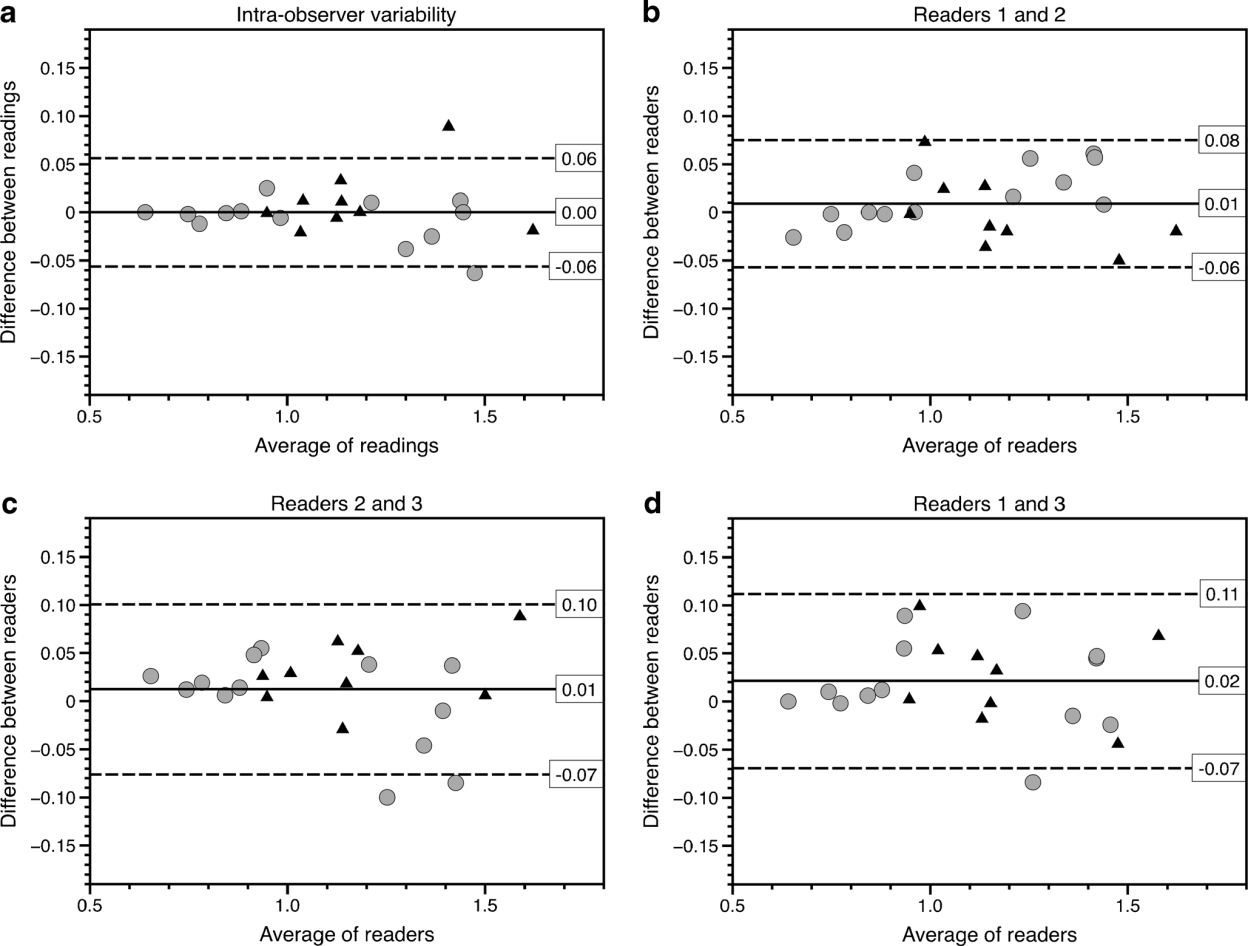
Median intra- and interobserver variability for whole-tumour apparent diffusion coefficient (ADC) measurements. **a**–**d** Bland–Altman plots show median ADC values for intra-observer variability (**a**), for observers 1 and 2 (**b**), for observers 2 and 3 (**c**) and for observers 1 and 3 (**d**). X-axis shows the average of the two readings, whereas the y-axis represents the difference between the readings. The *solid lines* represent the average absolute difference between the two readings. The *dashed lines* represent the 95% confidence intervals of the average differences (limits of agreement). The *grey circles* represent the measurements of the lesions before treatment; the *black triangles* represent the measurements of the post-treatment lesions. The Bland–Altman plots show narrow limits of agreement and somewhat larger disagreement at higher ADC values, but no definite bias. The outlier (patient 10, with average of readings of 1.45 × 10^−3^ mm^2^/s) in the intra-observer plot (**a**) was probably related to post-treatment haemorrhagic/necrotic changes. The outliers (patient 22, 1.25 × 10^−3^ mm^2^/s) in (**c**) and (**d**) represent measurements of the same relatively small lesion (7 cm^3^), which was difficult to discern from the surrounding renal tissue

**Fig. 4:**
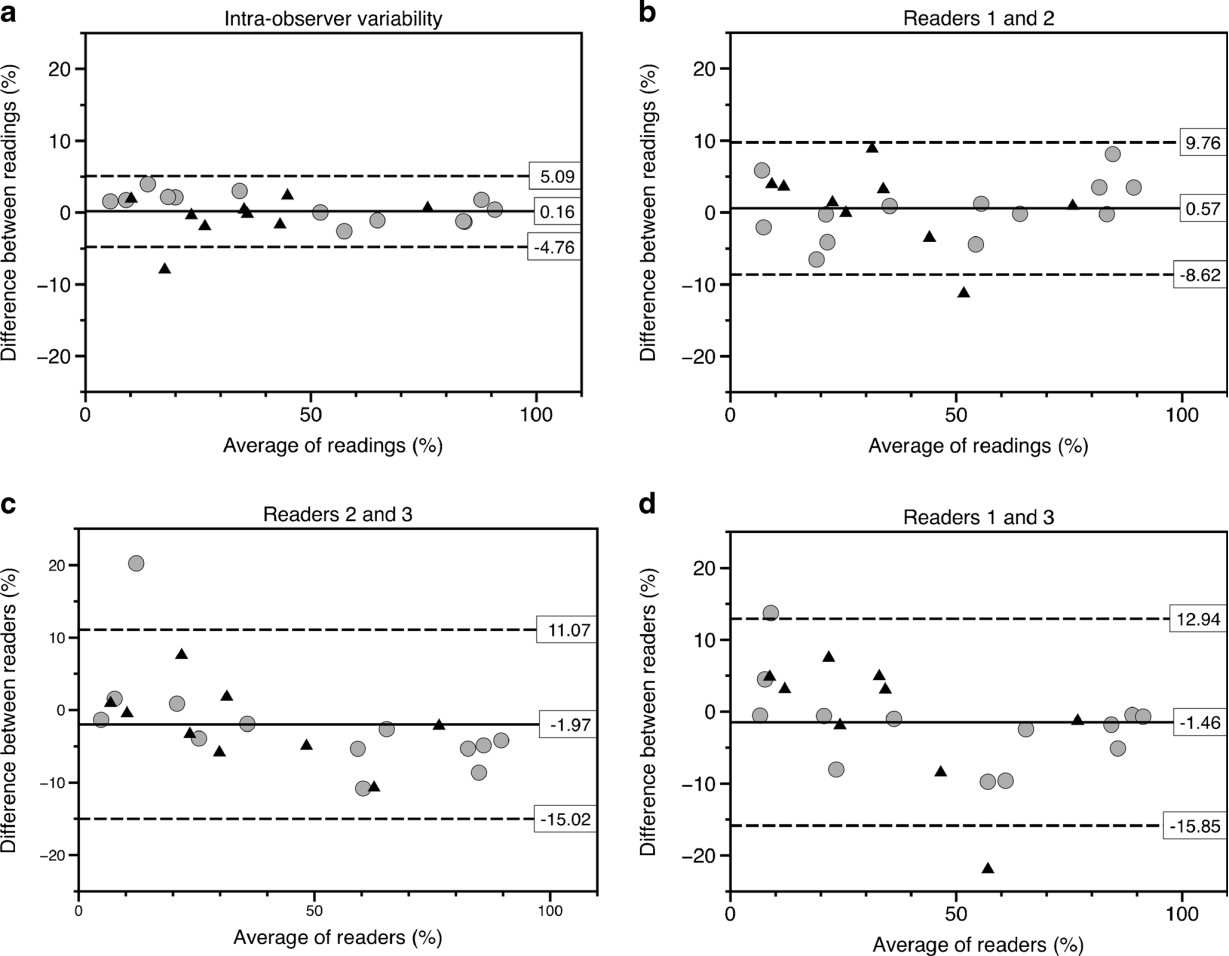
Intra- and interobserver variability for whole-tumour apparent diffusion coefficient (ADC) measurements. **a**–**d** Bland–Altman plots show the percentage of pixels below 1.00 × 10^−3^ mm^2^/s for intra-observer variability (**a**), for observers 1 and 2 (**b**), for observers 2 and 3 (**c**) and for observers 1 and 3 (**d**). The *grey circles* represent the measurements of the pretreatment lesions; the *black triangles* represent the measurements of the post-treatment lesions. The wide limits and the possible trend suggest that the percentage of low pixel measurements is a less-reliable measurement. **a** The outlier (patient 10, with average of readings of 17.6%) in the intra-observer plot is probably related to post-treatment haemorrhagic/necrotic changes. **b**, **d** The outliers (patient 54, average percentage of readings about 55%) represent a relatively small (6 cm^3^) post-treatment lesion. **c** The outlier (patient 22, average percentage of readings 12%) represents a relatively small lesion (7 cm^3^)

**Fig. 5:**
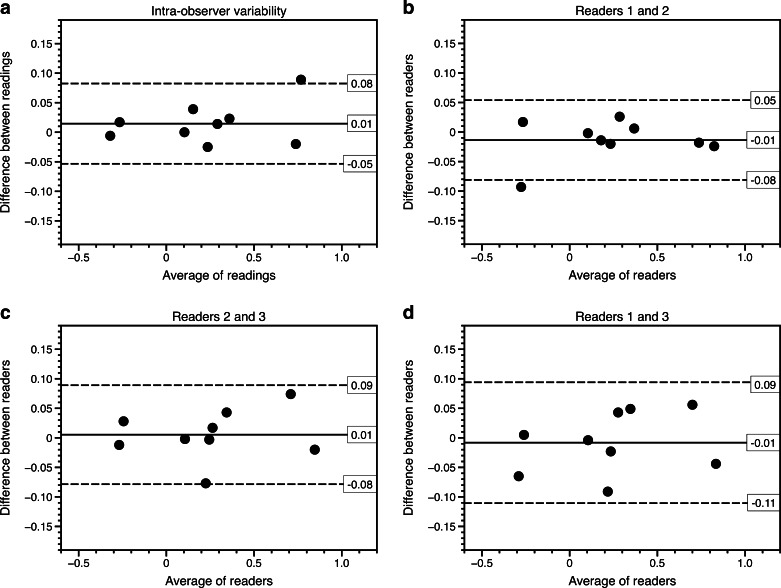
Intra- and interobserver variability for whole-tumour measurements of apparent diffusion coefficient (ADC) shift from pretreatment to post-treatment. **a**–**d** Bland–Altman plots show post-treatment median ADC minus pretreatment median ADC for intra-observer variability (**a**), for observers 1 and 2 (**b**), for observers 2 and 3 (**c**) and for observers 1 and 3 (**d**). **a** The outlier in the intra-observer plot represents the lesion with post-treatment changes as previously described (patient 10, average of readings of 0.77 × 10^−3^ mm^2^/s). The single outlier for the inter-observer analyses represents a relatively small lesion (patient 22, volume 7 cm^3^, average of readings of 0.22 × 10^−3^ mm^2^/s)

The intra-observer variability for whole-tumour ADC measurements is displayed in Figs. 3, 4 and 5. The Bland–Altman plot for median ADC demonstrates narrow limits of agreement (mean difference: 0; 95% limits of agreement: −0.06–0.06 × 10^−3^ mm^2^/s) indicating very low intra-observer variability. There was no striking trend to suggest a bias in the variability. One outlier represents a post-treatment lesion with haemorrhagic changes. The intra-observer variability was not significantly different for pre- or post-chemotherapy measurements (*P* = 0.31). The concordance correlation coefficient (ρ-c = 0.994 for median ADC; ρ-c = 0.995 for post-treatment minus pre-treatment median ADC; ρ-c = 0.996 for % low-ADC pixels) indicated very good agreement for all three tested parameters (Table 3).

**Table 3:** Concordance correlation coefficient for all parameters in intra- and interobserver variability

	Parameter	Coefficient of concordance	95% confidence interval
Intra-observer	Median ADC	0.994	0.986–0.998
	% low-ADC pixels	0.996	0.991–0.998
	Post-pre ADC	0.995	0.980–0.999
Interobserver	Median ADC, Observers 1-2	0.991	0.979–0.996
	Median ADC, Observers 2, 3	0.984	0.963–0.993
	Median ADC, Observers 1, 3	0.982	0.957–0.992
	% low-ADC pixels, Observers 1, 2	0.986	0.966–0.993
	% low-ADC pixels, Observers 2, 3	0.973	0.945–0.987
	% low-ADC pixels, Observers 1, 3	0.968	0.931–0.985
	Post-pre ADC, Observers 1, 2	0.995	0.980–0.999
	Post-pre ADC, Observers 2, 3	0.990	0.959–0.998
	Post-pre ADC, Observers 1, 3	0.993	0.971–0.998

*ADC* apparent diffusion coefficient, *Post*-*pre* post-treatment ADC values minus pretreatment values

### Interobserver variability

For median ADC (Fig. 3) the limits of agreement were narrow for all three reader pairs, indicating low interobserver variability (ranging −0.08–0.11 × 10^−3^ mm^2^/s). The average difference was close to zero and there was no suggestion of any bias in the variability, i.e. the variability was comparable between tumours with high and low ADC values.

For percentage of low-ADC pixels, Bland–Altman plots showed wider limits of agreement (−15.9–12.9%) with a trend towards negative differences between readers 2 and 3 and readers 1 and 3, indicating that reader 3 measured a higher percentage of low pixels in tumours with predominantly low ADC (Fig. 4). This linear relation was confirmed by a Spearman’s rho of −0.53 (*P* = 0.01) for readers 1 and 3 and Spearman’s rho of −0.66 (*P* = 0.01) for readers 2 and 3, indicating a negative relation between the mean ADC and the differences between the readers, suggesting a systematic error.

For the difference in median ADC before and after pre-operative therapy (Fig. 5), the limits of agreement ranged −0.11–0.09 × 10^−3^ mm^2^/s, indicating reasonably good interobserver agreement. The average difference was close to zero, and there was no suggestion of any bias in the variability.

The calculated correlation coefficient for all three parameters indicated good agreement for all tested parameters (Table 3).

The Wilcoxon rank sum test showed no statistically significant difference in inter-rater variability for pre- and post-treatment lesions (raters 1 and 2: *P* = 0.21; raters 2 and 3: *P* = 0.51; raters 1 and 3: *P* = 0.77).

The outliers represent either small lesions or lesions with relatively large haemorrhagic/necrotic or cystic components (Table 4). The outliers in all three intra-observer plots represent measurements of a single lesion that demonstrated haemorrhagic/necrotic changes after treatment. The outliers for the interobserver readings were almost all related to the relatively small size of the lesions.

**Table 4:** Characteristics of outliers identified in the intra- and interobserver variability analyses

Outlier	Comparison of interest	Patient number, pre/post treatment	Possible cause(s) for high variability
1	Intra, median ADC	10, post	Haemorrhagic/necrotic components
2	Inter, median ADC, readers 2 and 3	22, pre	Small (7 cm^3^), difficult to discern from normal renal tissue
3	Inter, median ADC, readers 2 and 3	8, pre	Cystic components (volume 17 cm^3^)
4	Inter, median ADC, readers 1 and 3	22, pre	Small (7 cm^3^)
5	Intra, post minus pre	10	Haemorrhagic/necrotic components post-treatment
6	Inter, post minus pre, readers 1 and 2	53	Post-chemotherapy lesion volume 8 cm^3^
7	Intra, % low-ADC pixels	10, post	Haemorrhagic/necrotic components
8	Inter, % low-ADC pixels, readers 1 and 2	54, post	Small (6 cm^3^)
9	Inter, % low-ADC pixels, readers 2 and 3	22, pre	Small (7 cm^3^)
10	Inter, % low-ADC pixels, readers 1 and 3	54, post	Small (6 cm^3^)

*ADC* apparent diffusion coefficient, *Inter* inter-rater, *Intra* intra-rater, *Post* post-treatment, *Pre* pretreatment

## Discussion

Our results show reasonably good intra- and interobserver agreement for whole-tumour median ADC and for chemotherapy-induced shift in median ADC. However, the estimates of percentage of low-ADC pixels seemed less reliable because the limits of agreement were wider and because there was an overall bias in the reader/reading differences. In other words, the latter variable seemed more sensitive to the definition of region of interest and the chosen threshold of subtraction, resulting in a wider 95% limit of agreement for all rater pairs. The percentage of low-ADC pixels, therefore, seems to be an inferior variable that we will not discuss further.

What is acceptable variability depends on the clinical application, the range of the true values and the degree of clinically relevant change in ADC measurements. McDonald et al. [12] reported a change in ADC values in six patients with nephroblastoma varying from 0.03 × 10^−3^ mm^2^/s to 0.81 × 10^−3^ mm^2^/s. Interestingly, those with necrosis or stromal differentiation all had an ADC shift of more than 0.14 × 10^−3^ mm^2^/s. We found that differences of approximately 0.10 × 10^−3^ mm^2^/s or more were reliably identified. This implies that ADC shifts reported by McDonald et al. [12] in regressing or stromally differentiating nephroblastoma are likely to be reliably identified. On the other hand, the reported ADC shift in nephroblastomas that respond with epithelial differentiation (≤0.08 × 10^−3^ mm^2^/s) is in the range of measurement error.

To our knowledge this is a unique study reporting reliability in ADC-distribution parameters in enhancing parts of nephroblastomas. Previous studies have highlighted the improved inter-rater variability for the whole-lesion ADC measurements for assessing the central value of the ADC distribution in different kinds of tumours [13–15]. Single-slice or sample ROI measurements are probably suboptimal in nephroblastoma because of the heterogeneity of these tumours, i.e. variable fractions of blastemal, epithelial and stromal cells. Under-sampling could conceal information that is important for response prediction and assessment [1, 16]. The second important component of our proposed method is the exclusion from ADC analysis all areas with little or absent gadolinium enhancement. Although the presence and extent of necrosis could serve as a biomarker for prediction and response assessment, in our experience necrotic areas in nephroblastomas sometimes demonstrate very low ADC values, mimicking highly cellular portions of the lesion. A possible explanation for low ADC in necrotic areas may be the pattern of chemotherapy-induced change described at histopathology, including coagulative-type necrosis, fibrosis, hemosiderin-laden macrophages and haemorrhage [17]. These haemorrhagic components result in susceptibility artefacts and incorrect ADC calculations that skew the ADC median values to lower values. We do not claim that simple thresholding provides a perfect mask for necrosis, but it is a pragmatic and easily achievable method for reducing the influence on the central ADC parameter of extreme values within necrotic regions. It may be argued that it is unjustified to exclude intratumoural regions from analysis, and that this introduces undesirable subjectivity to the analyses. However, knowing whether the ADC values within a cystic or necrotic component are high or low does not add diagnostic value.

Our retrospective study has several limitations. First, the number of subjects and number of lesions were limited, reflecting the low incidence of this tumour. Second, small and predominantly cystic tumours were excluded from final analysis. However, in daily clinical practise nephroblastomas are generally large lesions at presentation, and the solid components are the areas of interest for response prediction and assessment. Third, our proposed approach of whole-tumour ADC assessment is more time-consuming compared to the single-slice or sample ROI measurements that are quick and easy to perform. In addition, our method of measurements could be more highly influenced by the skills of the reader because several successive steps are required in our technique. However our results show good intra- and inter-rater variability. Fourth, we used the enhancement of erector spinae muscles as a threshold filter for excluding less-enhancing portions of the lesions. Formal perfusion analysis to appoint the subtraction threshold could further improve the selection of viable areas of the lesions. However, potential mismatch caused by slight movement between the diffusion, pre- and post-contrast acquisitions is difficult to resolve. Last, the degree of interobserver variability in measuring percentage of low ADC is not independent of the chosen threshold. However, some threshold had to be determined, and based on our experience we chose the threshold of 1.00 × 10^−3^ mm^2^/s [12].

## Conclusion

Estimation of the median of the ADC distribution in enhancing parts of nephroblastoma is reliable to within approximately 0.1 × 10^−3^ mm^2^/s. Chemotherapy-induced shifts in median ADC of about 0.1 × 10^−3^ mm^2^/s or more are therefore unlikely to be caused by random error. This is encouraging because reported shifts in ADC values in regressive and stromally differentiating nephroblastomas are larger than this threshold.
